# Usefulness of arterial spin labeling perfusion as an initial evaluation of status epilepticus

**DOI:** 10.1038/s41598-021-03698-7

**Published:** 2021-12-20

**Authors:** Tae-Joon Kim, Jin Wook Choi, Miran Han, Byung Gon Kim, Sun Ah Park, Kyoon Huh, Jun Young Choi

**Affiliations:** 1 Department of Neurology, Ajou University School of Medicine, Suwon, Republic of Korea; 2 Department of Radiology, Ajou University School of Medicine, Suwon, Republic of Korea; 3 Departments of Brain Science and Neurology, Ajou University School of Medicine, 164, World cup-ro, Yeongtong-gu, Suwon, Gyeonggi-do 16499 Republic of Korea; 4 Department of Anatomy, Ajou University School of Medicine, Suwon, Republic of Korea; 5 Department of Medical Humanities & Social Medicine, Ajou University School of Medicine, Suwon, Republic of Korea

**Keywords:** Diseases, Neurology

## Abstract

This study aimed to evaluate the sensitivity and prognostic value of arterial spin labeling (ASL) in a large group of status epilepticus (SE) patients and compare them with those of other magnetic resonance (MR) sequences, including dynamic susceptibility contrast (DSC) perfusion imaging. We retrospectively collected data of patients with SE in a tertiary center between September 2016 and March 2020. MR images were visually assessed, and the sensitivity for the detection of SE and prognostication was compared among multi-delay ASL, DSC, fluid-attenuated inversion recovery (FLAIR), and diffusion-weighted imaging (DWI). We included 51 SE patients and 46 patients with self-limiting seizures for comparison. Relevant changes in ASL were observed in 90.2% (46/51) of SE patients, a percentage higher than those for DSC, FLAIR, and DWI. ASL was the most sensitive method for initial differentiation between SE and self-limiting seizures. The sensitivity of ASL for detecting refractory SE (89.5%) or estimating poor outcomes (100%) was higher than those of other MR protocols or electroencephalography and comparable to those of clinical prognostic scores, although the specificity of ASL was very low as 9.4% and 15.6%, respectively. ASL showed a better ability to detect SE and predict the prognosis than other MR sequences, therefore it can be valuable for the initial evaluation of patients with SE.

## Introduction

Status epilepticus (SE) is a common neurological emergency manifesting as a prolonged seizure or multiple seizures without baseline recovery^1^. The high overall mortality of about 15% highlights the importance of immediate diagnosis, treatment, and prognostic assessment^2^. To evaluate patients with SE, electroencephalography (EEG) and brain imaging are essential tools. EEG is necessary for SE diagnosis and monitoring and has been identified as an important tool for determining patient prognosis^3^. Magnetic resonance image (MRI) plays a significant role in the initial assessment of etiology; however, its value in ascertaining the diagnosis or prognosis of patients with SE remains unclear^4^.

Arterial spin labeling (ASL), a noninvasive perfusion imaging MRI technique using magnetically labeled water in the blood as an endogenous tracer, has recently been reported to aid in the diagnosis and localization of seizures or SE^5^. In self-limiting focal seizures, ASL shows a sensitivity of 50–74% for identifying the seizure focus, and the usual direction of change in ASL was indicative of hypoperfusion, which can be explained by neurological dysfunction in the postictal period^6–9^. Two previous studies assessed the sensitivity of ASL in small groups of patients with SE and compared this method with diffusion-weighted imaging (DWI). ASL identified focal hyperperfusion in 13 of 20 SE patients in the peri-ictal state (65%)^10^ and identified ictal hyperperfusion in all 15 nonconvulsive SE (NCSE) patients, demonstrating the superiority of ASL over DWI^11^. Moreover, there was a tight topographical relationship between the localization of ictal ASL findings and the epileptogenic lesion corresponding to EEG abnormalities. In addition, a recent study using ASL images showed that thalamic hyperperfusion in 28 patients with suspected NCSE was particularly correlated with NCSE diagnosis and rhythmic activities of EEG with high accuracy^12^. Thus, ictal hyperperfusion is useful for the diagnosis and localization in SE, and ASL can be a sensitive perfusion MRI technique for detecting ictal changes. So far, no comprehensive study has examined the sensitivity of ASL in SE patients compared to those of other perfusion-weighted MRI techniques such as dynamic susceptibility contrast (DSC). DSC perfusion imaging uses an external contrast agent like gadolinium and is typically included in magnetic resonance (MR) sequences for SE patients^5,13,14^. As ASL has the advantage over DSC of being noninvasive and easily repeatable due to the unnecessity of gadolinium, replacing DSC with ASL could be beneficial.

In the present study, we investigated the usefulness of ASL as a diagnostic measure and the relation of ASL to refractoriness and outcomes in a large population of patients with SE. For ASL, the latest multi-delay method was used to provide the best accuracy. Cerebral blood flow (CBF) measured by ASL and relative CBF measured by DSC were compared. Specifically, we aimed to evaluate the advantages of ASL over DSC, DWI, fluid-attenuated inversion recovery (FLAIR), and EEG, which constitute the initial and essential diagnostic tools used to assess patients with SE.

## Results

During the study period, 195 patients were diagnosed with SE, out of which 65 underwent brain MRI with ASL, as well as continuous EEG monitoring. Eight patients did not meet the inclusion criteria regarding time differences between symptoms and ASL, and six patients were excluded from subsequent data analyses due to severe MRI artefacts: two with insufficient labeling, two with a metallic artefact, one with an active structural lesion that could affect the interpretation of ASL, and one with severe motion artefacts. Thus, the final analysis included 51 patients.

### Clinical characteristics of SE patients

The mean age of the 51 patients with SE was 62.5 ± 19.1 years, and 35 (68.6%) were male. Their clinical characteristics are summarized in Table 1. Twenty (39.2%) patients had epilepsy, and five experienced a previous SE event. Remote symptomatic etiology (41.2%) was the most frequent cause, followed by the acute and progressive symptomatic etiologies (21.6% and 19.6%, respectively). Most cases (78.4%) involved tonic–clonic SE as the presenting symptom. SE was refractory in 19 cases (37.3%), of which 5 were super-refractory cases requiring induced therapeutic coma. EEG signs of ictal activities, epileptiform discharges, or postictal regional slowing were observed in 43 cases (84.3%), whereas the remaining 8 cases involved normal EEG findings or intermittent nonspecific slowing.

**Table 1:** Clinical characteristics of 51 patients with status epilepticus.

Item	Mean ± SD or number (%)
**Demographic factors**	
Age	62.5 ± 19.1
Sex (male)	35 (68.6%)
Previous histories related to seizure	
Epilepsy	20 (39.2%)
SE history	5 (9.8%)
**SE characteristics**	
Etiology	
Acute symptomatic	11 (21.6%)
Remote symptomatic	21 (41.2%)
Progressive symptomatic	10 (19.6%)
Idiopathic/cryptogenic	5 (9.8%)
AED withdrawal	4 (7.8%)
Dynamics of SE	
Tonic–clonic SE only	27 (52.9%)
NCSE only	4 (7.8%)
Tonic–clonic SE evolving into NCSE	13 (25.5%)
Focal SE	4 (7.8%)
Focal SE evolving into Tonic–clonic SE	3 (5.9%)
Characteristic EEG type	
Ictal pattern	20 (39.2%)
Ictal-interictal continuum	8 (15.7%)
Interictal spike or regional slowing	15 (29.4%)
Normal or intermittent generalized slowing	8 (15.7%)
Refractoriness	
Non-RSE	32 (62.7%)
RSE (not super-RSE)	14 (27.5%)
Super-RSE	5 (9.8%)
**Time variable**	
Time between FAT and ASL (hours)	10.8 ± 7.7
Time between benzodiazepine and ASL (hours)	7.4 ± 5.8
Time between ASL and EEG (hours)	7.7 ± 16.0
**MRI detection**	
ASL: hyper- or hypo-perfusion	46 (90.2%)
Hyperperfusion	42 (82.4%)
Thalamic, any change	25 (49.0%)
Thalamic ipsilateral coincidence	20 (39.2%)
DSC: hyper- or hypo-perfusion	20 (39.2%)
FLAIR: signal change due to SE	27 (52.9%)
DWI: diffusion restriction due to SE	18 (35.3%)
**Treatments**	
Coma therapy	5 (9.8%)
Treated on ICU	36 (70.6%)
Tracheal intubation	15 (29.4%)
Days on ICU	9.7 ± 18.3
Hospital days	20.0 ± 19.6
**Prognosis**	
mRS, premorbid (median [IQR])	2 [1–3]
mRS, at discharge (median [IQR])	2 [2–4]
STESS (median [IQR])	3 [2–5]
EMSE	57.1 ± 34.3
Poor outcome (mRS ≥ 4)	19 (37.3%)

*SD* standard deviation, *SE* status epilepticus, *AED* antiepileptic drug, *NCSE* nonconvulsive SE; *EEG* electroencephalography, *RSE* refractory status epilepticus, *FAT* first abnormal time, *ASL* arterial spin labeling, *MRI* magnetic resonance imaging, *DSC* dynamic susceptibility contrast, *FLAIR* fluid attenuated inversion recovery, *DWI* diffusion weighted imaging, *ICU* intensive care unit, *mRS* modified Rankin Scale, *IQR* interquartile range, *STESS* status epilepticus severity score, *EMSE* epidemiology-based mortality score in status epilepticus.

Of the 51 patients, 43 had MRI before EEG, and 8 had MRI after the start of EEG, of which 5 had ictal EEG. Before the MRI scan, 50 patients except one were administered intravenous benzodiazepine. Forty patients underwent MRI after second-line anti-epileptic drug administration, and four patients underwent MRI after starting continuous anesthetic drugs. Detailed data, such as the time delay between EEG and MRI, can be found in the Supplementary Table S1.

Most patients (70.6%) were treated in intensive care units, and 15 of those required tracheal intubation. Approximately one-third (37.3%) of all patients had a poor outcome (mRS score ≥ 4) at discharge.

### Usefulness of ASL in SE diagnosis

Inter-rater reliability showed substantial to near-perfect agreement in the visual MR assessment^15^. For ASL images, Cohen’s kappa coefficient was 0.811 between the two raters. The respective coefficients for DSC, FLAIR, and DWI were 0.761, 0.922, and 0.783, respectively.

**Figure 1:**
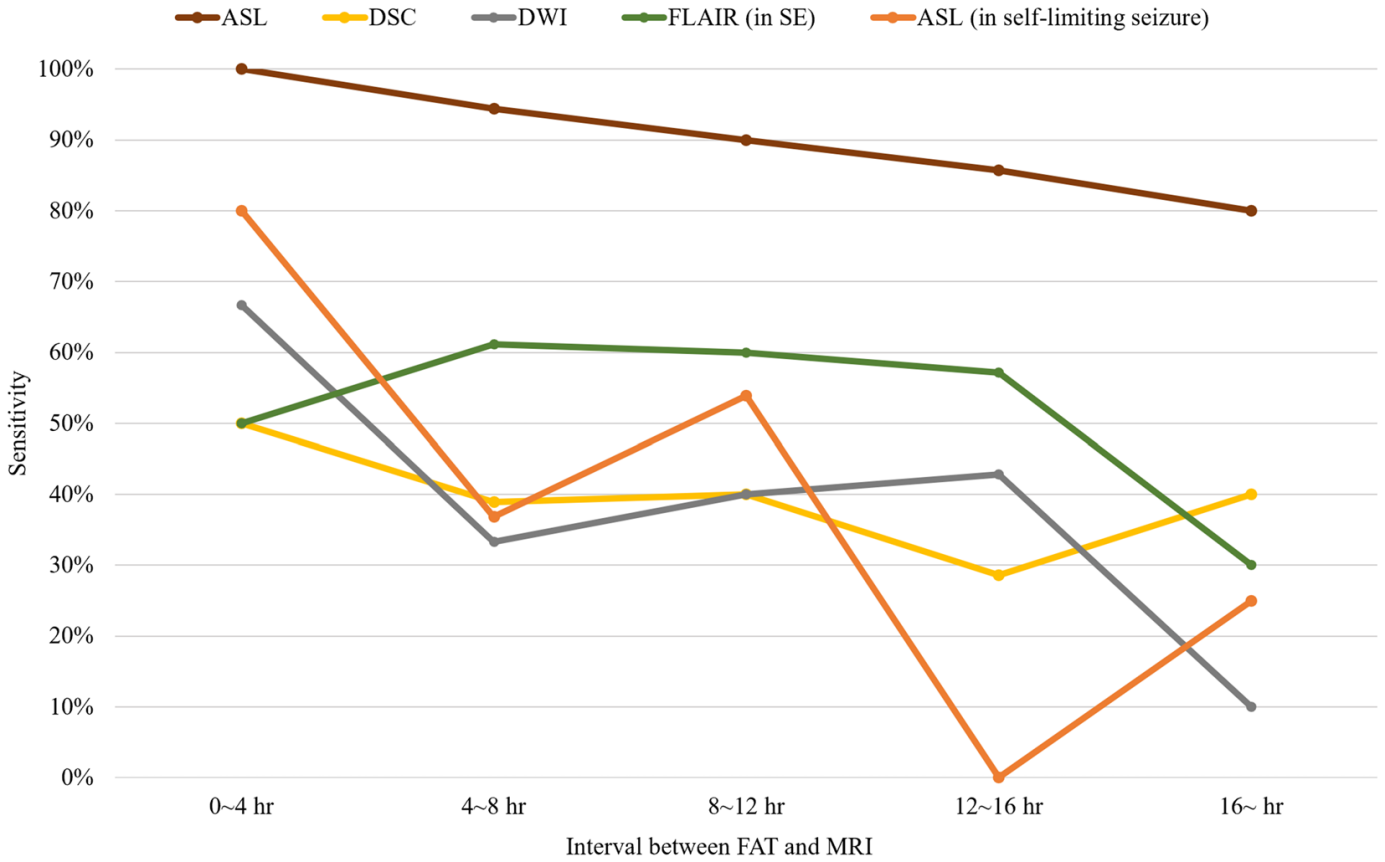
Comparison of the sensitivities of MR sequences according to the interval between the first abnormal time and MRI acquisition. Generally, the sensitivity of each sequence decreases over time. ASL has the highest sensitivity for detecting status epilepticus in all time intervals compared to other sequences. The tendency of decreasing sensitivity with time is also seen for self-limiting seizures. For self-limiting seizures, the interval between the last seizure and MRI acquisition was used. *ASL* arterial spin labeling, *DSC* dynamic susceptibility contrast, *DWI* diffusion-weighted imaging, *FAT* first abnormal time, *FLAIR* fluid-attenuated inversion recovery, *MR* magnetic resonance, *MRI* magnetic resonance imaging, *SE* status epilepticus.

Among the four MR sequences, changes were most frequently detected on ASL (Table 1). ASL showed altered perfusion in 90.2% (46/51) of patients, whereas DSC, FLAIR, and DWI revealed SE-related changes in 39.2%, 52.9%, and 35.3% of patients, respectively. Of the 46 patients showing changes on ASL, 42 showed hyperperfusion, and 4 presented with hypoperfusion. Thalamic perfusion abnormality in ASL was observed in approximately a half (49.0%) of SE patients and most of them (20/25) had ipsilateral coincidence with cortical ASL change. We then compared ASL hyperperfusion with suspected seizure onset zone from EEG. Among the 42 patients showing hyperperfusion on ASL, 34 had localizable EEG of which 31 matched according to the lateralization and 26 matched in lobar level (Supplementary Table S1). Moreover, ASL showed better sensitivity for localizing SE-related changes than other imaging protocols, including DSC (*P* < 0.001). In addition, ASL showed superior sensitivity over all other MR sequences, and sensitivity tended to decrease gradually as MR was taken later from the time of seizure onset Fig. 1.

### ASL in self-limiting seizures versus SE

The changes in ASL sensitivity for SE may be caused by SE itself or seizure-related changes. Hence, we examined the MR images of 46 patients with self-limiting seizures and compared them to those of patients with SE. The mean age in this non-SE group was 39.4 ± 16.7 years, and 29 patients were male. Only 19 (41.3%) of the 46 patients showed seizure-related perfusion changes on ASL, and nearly half (8/19, 42.1%) of them showed hypoperfusion. Among the 19 patients with changes on ASL, 68.4% (13/19) experienced at least two seizures in a cluster before MRI acquisition; contrastingly, only 29.6% (8/27) of patients without changes on ASL experienced at least two seizures (*P* = 0.016). Furthermore, these patients tended to have less time between the last seizure and MRI acquisition than patients without changes on ASL (7.6 ± 4.3 vs. 10.1 ± 5.7 h, *P* = 0.233). Except for one individual, all patients with self-limiting seizures showed changes on ASL within 0–12 h from the last seizure.

The accuracy of each MR protocol in distinguishing SE from self-limiting seizures was evaluated, and ASL was the most sensitive and accurate method (Table 2). The sensitivity and accuracy values of ASL for detecting SE were 90.2% and 75.3%, respectively; DWI had the highest specificity (97.8%). Notably, thalamic ASL change showed higher sensitivity for distinguishing SE from self-limiting seizure than other MR sequences.

**Table 2:** Sensitivity, specificity, PPV, NPV, and accuracy of each method with SE versus self-limiting seizure.

	Sensitivity (%)	Specificity (%)	PPV (%)	NPV (%)	Accuracy (%)
**Differentiating SE (n = 51) from self-limiting seizure (n = 46)**					
ASL, any change	90.2	58.7	70.8	84.4	75.3
ASL, only hyperperfusion	79.2	79.5	82.4	76.1	79.4
Thalamic ASL, any change	83.3	61.2	49.0	89.1	68.0
Thalamic ASL, ipsilateral	87.0	58.1	39.2	93.5	64.9
DSC	39.2	82.6	71.4	55.1	59.8
FLAIR	52.9	91.3	87.1	63.6	71.1
DWI	35.3	97.8	94.7	57.7	64.9

*PPV* positive predictive value, *NPV* negative predictive value, *SE* status epilepticus, *ASL* arterial spin labeling, *DSC* dynamic susceptibility contrast, *FLAIR* fluid attenuated inversion recovery, *DWI* diffusion weighted imaging.

### Sensitivity of ASL for assessing the refractoriness and prognosis of SE

As many SE patients with uncontrolled seizures and poor outcomes showed changes on ASL, we speculated that these changes were associated with the refractoriness and prognosis of SE. To address this issue, we evaluated each MR sequence, clinical scores, and EEG signs for patients with refractory SE or poor outcomes. ASL showed remarkable sensitivity (89.5%) in identifying refractory SE, although the specificity was low (9.4%; Table 3). Moreover, ASL had the highest sensitivity (100.0%) for estimating poor outcomes compared to other imaging sequences and EEG, and its sensitivity was comparable to those of conventional prognostic scores. ASL was also useful in predicting ictal activity or ictal-interictal continuum on EEG. When limited to hyperperfusion, the hyperperfusion change of ASL showed similar sensitivity and slightly higher accuracy compared to all changes of ASL. Interestingly, thalamic changes had modest specificity (61.5%, 69.2%) along with low sensitivity (36.0%, 44.0%) predicting refractoriness or poor outcome, however, the opposite applied in the case of predicting EEG activity. Besides, DWI was the most specific MR sequence for refractoriness and poor outcomes, with specificity values of 75.0% for both measures. The results of each method for refractory SE, poor outcomes, and seizure or ictal-interictal continuum on EEG are summarized in Table 3.

**Table 3:** Sensitivity, specificity, PPV, NPV, and accuracy of each method with prognosis.

	Sensitivity (%)	Specificity (%)	PPV (%)	NPV (%)	Accuracy (%)
**(Super-)RSE (n = 19) or not (n = 32)**					
ASL, any^a^	89.5	9.4	37.0	60.0	39.2
ASL, hyper^a^	89.5	21.9	40.5	77.8	47.1
Thalamic, any	36.0	61.5	47.4	50.0	49.0
Thalamic, ipsi	30.0	58.1	31.6	56.3	47.1
DSC	36.8	59.4	35.0	61.3	51.0
FLAIR	63.2	53.1	44.4	70.8	56.9
DWI	52.6	75.0	55.6	72.7	66.7
**Poor outcome (mRS ≥ 4; n = 19) or not (n = 32)**					
ASL, any^a^	100.0	15.6	41.3	100.0	47.1
ASL, hyper^a^	94.7	25.0	42.9	88.9	51.0
Thalamic, any	44.0	69.2	57.9	56.3	56.9
Thalamic, ipsi	45.0	67.7	47.4	65.6	58.8
DSC	52.6	68.8	50.0	71.0	62.7
FLAIR	57.9	50.0	40.7	66.7	52.9
DWI	52.6	75.0	55.6	72.7	66.7
EEG	84.2	15.6	37.2	62.5	41.2
STESS (≥ 4)	73.7	71.9	60.9	82.1	72.5
STESS (≥ 3)	89.5	50.0	51.5	88.9	64.7
EMSE (> 55)	84.2	65.6	59.3	87.5	72.5
EMSE (> 35)	100.0	53.1	55.9	100.0	70.6
**Ictal activities + IIC (n = 28) or not (n = 23)**					
ASL, any^a^	92.9	13.0	56.5	60.0	56.9
ASL, hyper^a^	92.9	30.4	61.9	77.8	64.7
Thalamic, any	64.0	53.8	57.1	60.9	58.8
Thalamic, ipsi	60.0	48.4	42.9	65.2	52.9
DSC	50.0	73.9	70.0	54.8	60.8
FLAIR	50.0	43.5	51.9	41.7	47.1
DWI	46.4	78.3	72.2	54.5	60.8

^a^Any, any change including both hyper- and hypo-perfusion; hyper, only hyperperfusion.

*PPV* positive predictive value, *NPV* negative predictive value, *RSE* refractory status epilepticus, *ASL* arterial spin labeling, *DSC* dynamic susceptibility contrast, *FLAIR* fluid attenuated inversion recovery, *DWI* diffusion weighted imaging, *mRS* modified Rankin Scale, *EEG* electroencephalography, *STESS* status epilepticus severity score, *EMSE* epidemiology-based mortality score in status epilepticus, *IIC* ictal-interictal continuum.

### Comparison of quantitative change of ASL

Because all comparisons were performed with qualitative data by visual analysis, it is necessary to check whether visual analysis reflects quantitatively changes in ASL. Notably, hyperperfusion group either SE or single seizure showed significantly higher asymmetric index in quantitative analysis than that of the group without ASL changes. Although there was no statistically significance, ASL change SE group showed the highest changes in quantitative analysis. Supplementary Fig. 1 showed comparison of quantitative analysis of ASL in each group.

## Discussion

This retrospective study investigated the usefulness of multiple neuroimaging techniques for detecting ictal or peri-ictal states in 51 patients with SE and revealed that the most prominent and frequent changes were seen on ASL, compared to other MR sequences and EEG. Our findings demonstrated that ASL, among all MRI modalities, differentiated SE from self-limiting seizures with the highest accuracy. Furthermore, in patients with ictal or post-ictal SE, ASL showed the highest sensitivity in estimating poor outcomes or the refractoriness of SE. Particularly, ASL was significantly more sensitive than other MR sequences. These results indicate that ASL may differentiate SE from self-limiting seizures and is the most sensitive prognostic tool for patients with SE. Therefore, this study shows that ASL can be an important diagnostic modality in the initial evaluation of SE.

We compared the usefulness of ASL with that of other MR methods in terms of diagnosis and prognosis in a large group of patients with SE. ASL is an MR technique involving brain perfusion that measures regional CBF and cortical activation, and various applications have been established^16^. ASL has been studied in self-limiting seizures and shown to have moderate diagnostic sensitivity (60–79%)^6,7,17^. In our study, patients with self-limiting seizure showed focal perfusion changes less frequently (41.3%) on ASL compared to the previous studies, while ASL of patients with repeated seizures was more likely to show changes. This result suggests that seizure-related changes on ASL are closely related to the seizure burden before MRI. Compared to EEG, FLAIR, and DWI, ASL has demonstrated its usefulness in the diagnostic evaluation of patients with SE or some cases of self-limiting seizures.

During seizures, cerebral metabolic demands rapidly increase, raising cerebral blood flow^18^. In this respect, neuroimaging methods for detecting increased flow signals in the cerebral arteries of the affected cortical regions can assist in the diagnosis of SE, especially in cases of NCSE. Single-photon emission computed tomography (SPECT) can localize focal ictal hyperperfusion in SE even when the EEG findings are inconclusive^19–21^. but the feasibility of SPECT for SE patients in the emergency room might be limited. Computed tomography (CT) perfusion imaging can be feasible, and fairly high sensitivity of 77–79% for detecting focal cortical hyperperfusion in patients with SE or NCSE has been reported^22,23^. CT perfusion imaging is often readily available but cannot completely substitute MRI in evaluating and assessing SE because MRI is superior in detecting inflammatory changes or subtle structural abnormalities, resulting in a higher etiological diagnostic yield^24^.

In a recent study of 28 NCSE patients, thalamic hyperperfusion were confirmed in 57% of cases and showed particularly high accuracy in diagnosing NCSE^12^. In the same study, cortical hyperperfusion was observed in 86%. These results from the previous study were consistent with the observed frequencies of our research, and are in line with the fact that changes in thalamic perfusion was more sensitively associated to EEG ictal rhythm.

Why did ASL have the highest sensitivity for detecting SE among the various MR modalities and EEG? According to our results, the earlier the MR image was acquired, the higher the sensitivity of ASL was to detect SE and self-limiting seizures. The first metabolic or pathologic change in seizure, depending on electrical activity, is increased perfusion^25^. Therefore, perfusion imaging modalities, such as SPECT, successfully localize seizure-onset zones immediately after a seizure and can be used for pre-surgical evaluation. Long-term seizures cause metabolic and pathologic changes and subsequent alterations on DWI and FLAIR as well as perfusion images^5,25^. In this study, ASL was more sensitive than DSC, a conventional and widely used MR perfusion method, in patients with SE. In DSC, blood vessel permeability is a major confounder in relative CBF measurements and can affect the accuracy of findings near regions rich in blood vessels, such as the medial temporal lobe near the circle of Willis^26^. ASL is relatively insensitive to permeability changes because it uses a diffusible tracer, arterial water. Additionally, ASL benefits from an improved signal-to-noise ratio in high field strength MRI and improved pulse sequences and multichannel receiver array coils^27^. In SE, a group of patients with changes in ASL comprised all subjects with changes in DSC, which means superior sensitivity of ASL to that of DSC. In a few cases where the EEG could not localize the SE, ASL was useful in diagnosing SE. Therefore, ASL may have the highest detection rate among various initial evaluation methods currently available for SE.

ASL was also highly predictive of a poor outcome. The current prognostic strategy in SE is mainly based on scoring systems such as STESS (Status Epilepticus Severity Score) and EMSE (Epidemiology-based mortality score in status epilepticus), which rely on clinical parameters^3,28^. MRI-based prognosis prediction has been attempted using DWI and FLAIR, but the sensitivity was not high^29^. Brain imaging is necessary to identify the etiology, which is required for the EMSE. According to our results, adding ASL to MR protocols is feasible and can provide useful information for the diagnostic process in patients with SE. The high sensitivity of ASL suggests that ASL reflects the increase in perfusion due to neurovascular unit coupling in SE better than other MRI modalities. However, DWI had the highest specificity to determine a poor outcome in SE among the various MR techniques because DWI reflects neuronal damage secondary to SE^25,30^. Therefore, we believe that the combination of ASL and DWI can help predict SE outcomes and suggest that ASL and DWI should be essential sequences in the initial evaluation for SE.

Despite the high sensitivity, ASL perfusion change had a disadvantage of low specificity and accuracy. This result can be a major obstacle in interpreting the results of ASL. The specificity was particularly low in the prediction of prognosis including refractoriness and poor outcome because most of the SE patients accompanied perfusion change and the result was reflected in the ASL image. However, the low specificity of ASL can be overcome by combining it with DWI or thalamic ASL change, which has relatively high specificity. In comparison with self-limiting seizures, ASL showed a modest degree of specificity (58.7%) for SE. Additionally in some clinical practice such as NCSE or subtle SE, MRI might be taken before EEG, and abnormal findings in MRI can lead to more immediate management taking advantage of high sensitivity of ASL, which can help shorten the time to EEG or administer anti-epileptic drugs^12^. Considering the poor prognosis of refractory SE, appropriate and timely treatment can help improve the prognosis and reduce mortality.

This study has some limitations. First, results may differ depending on the employed ASL technique. Previous studies have used pulsed ASL sequences or pseudo-continuous ASL with a shorter post-labeling delay. This study had the advantage of using state-of-the-art multi-delay ASL, the most sensitive sequence^27^. However, this ASL method may be associated with time- and equipment-related limitations, preventing it from being commonly used in other centers. Second, this study used mainly qualitative approach by visual rating. However, considering that visual image assessment is a clinically plausible process in the acute stage of SE, it is meaningful that this study was based on visual rating. Furthermore, comparison among visually ASL changed group or non-changed group showed significantly difference of asymmetry index. This finding supports visual analysis alone would be enough to decide whether perfusion is changed or not. . Third, the two patient groups (SE and self-limiting seizures) differed substantially in age. It is known that neurovascular coupling decreases with age^31,32^. Therefore, our results may be explained by disease-related perfusion changes rather than other inter-group differences since SE-related hyperperfusion was more frequently observed in the SE group despite the older age of patients in this group. Lastly, there might be selection bias of patients with SE, as they were selected retrospectively at a tertiary center.

In summary, this study showed that ASL could facilitate diagnosis and prognostication in the acute phase of SE. Our results propose that ASL should be an essential modality in the initial evaluation of SE. Future prospective or direct comparative studies involving other perfusion methods, such as CT perfusion imaging, may help in further establishing the usefulness of ASL in patients with SE.

## Methods

### Participants

This study retrospectively reviewed the medical records of adult patients who had SE as a presenting symptom and were admitted to the Department of Neurology at a tertiary center between September 2016 and March 2020. SE was diagnosed according to the operational definition proposed by the International League Against Epilepsy^30^. The time point beyond which a seizure was regarded as a continuous seizure was 5 min for tonic–clonic SE, 10 min for focal SE with impaired consciousness. The diagnosis of NCSE was made according to the Salzburg criteria^33^. The present study included patients if (1) they were diagnosed with SE as already described, (2) had undergone continuous EEG monitoring, and (3) had undergone brain MRI with ASL within 24 h of symptom onset or within 1 day after experiencing a suspected ictal event, such as nonconvulsive or subtle SE. For comparison, patients admitted for self-limiting focal epileptic seizures and who underwent brain MRI with ASL within 24 h from the last seizure onset were enrolled as the non-SE group. This retrospective study was approved by the Institutional Review Board of Ajou University Hospital (IRB no. AJIRB-MED-MDB-20-114), and the requirement to obtain informed consent was waived. All methods in the present study were performed in accordance with the relevant guidelines and regulations.

### Clinical variables

Data about demographics, medical conditions, hospital course, SE characteristics, brain MRI, and EEG were collected. Medical conditions included a previous diagnosis of epilepsy or SE. SE characteristics included etiology, dynamics, refractoriness, and EEG results. Hospital course data included various time points (first abnormal time, benzodiazepine injection time, and ASL time), treatment details (coma therapy, intensive care unit stay, tracheal intubation, and hospital days), and prognosis (premorbid modified Rankin Scale [mRS] score, discharge mRS score, status epilepticus severity score [STESS], and epidemiology-based mortality score in status epilepticus [EMSE])^3,28^. An mRS score of 4 or higher indicated a poor outcome. The most accurate cut-off scores of STESS or EMSE were obtained by ROC curve.

EEG data included any available short-term or long-term recordings. Their interpretation was based on the 2012 version of the American Clinical Neurophysiology Society’s Standardized Critical Care EEG Terminology^34^. The EEG results were classified according to four grades: (1) ictal activities, (2) ictal-interictal continuum (including periodic discharges and regional rhythmic activities), (3) interictal spikes or postictal regional slowing, or (4) normal or intermittent nonspecific slowing^35,36^. In the presence of findings classified in the groups (1)–(3), the EEG could be used to diagnose and localize an SE.

### Brain MRI acquisition

Relevant brain images, including ASL images, were collected. The brain MR sequences included ASL, FLAIR, DWI, and DSC images. When taking MRI sequences, the time required for ASL was about 5 min, the time required for DSC was about 3 min, and the time interval between ASL and DSC was about 11 min. MRI was taken without overt convulsive movement.

Multi-delay ASL and DSC perfusion imaging were included in our imaging protocol for epilepsy patients who underwent brain MRI with a 3-T scanner (750w; GE Healthcare). The brain MRI protocol for epilepsy patients included pre- and post-contrast 3D T1-weighted fast spoiled gradient-echo imaging, 3D CUBE T2-weighted imaging (T2WI), axial T2WI, axial T2 FLAIR, DWI, gradient-echo imaging, multi-delay ASL, and DSC perfusion imaging.

DWI comprised single-shot echo-planar imaging sequences acquired in the axial plane with the following parameters^37^: repetition time (TR), 3000 ms; echo time (TE), 80 ms; b-value, 1000 s/mm2; field of view, 22 cm; matrix size, 128 × 128; and 28 slices with 5-mm slice thickness and without interslice gap. Apparent diffusion coefficient (ADC) values were calculated at two different b-values (b = 0 and b = 1000 s/mm2). DWI images and ADC maps were generated on the scanner console at the time of imaging.

Parameters for multi-delay ASL imaging were as follows: TR, 5904 ms; TE, 11.7 ms; field of view, 24 cm; 640 sampling points on 4 spirals (matrix size, 640 × 4); section thickness, 5 mm; and number of sections, 28. We used a multi-delay ASL sequence based on Hadamard encoding to obtain 7 perfusion-weighted images with different post-labeling delays and effective labeling durations. The post-labeling delay times were 1.00, 1.22, 1.48, 1.78, 2.15, 2.63, and 3.32 s. The effective labeling durations were 1.00, 1.22, 1.48, 1.78, 2.15, 2.63, and 3.32 s. Utilizing Hadamard encoding, CBF images from 7 different post-labeling delays could be generated from a single acquisition, and each CBF image was an average of 4 images.

DSC T2 ∗ -weighted images were acquired using a single-shot gradient echo-planar imaging sequence with the following parameters: TR, 2300 ms; TE, 27.9 ms; flip angle, 60°; field of view, 24 × 24 cm2; matrix, 128 × 128; slice thickness, 5 mm; and number of excitations, 1. Images were acquired after gadobutrol (Gadovist, Bayer HealthCare) injection (0.1 mmol/kg, flow rate 3 mL/s) using a power injector immediately followed by a 20-mL saline flush. Perfusion maps of relative CBF, relative cerebral blood volume, and time to peak were generated using the commercial software nordicICE (v4.2.0, https://nordicneurolab.com, NordicNeuroLab, Bergen, Norway). The measured tissue concentration–time curve was deconvolved. The arterial input function was identified in the automatic detection mode at the level of the middle cerebral artery, and the number of arterial input function pixels was 5.

### Brain MRI: visual rating and inter-rater reliability

**Figure 2:**
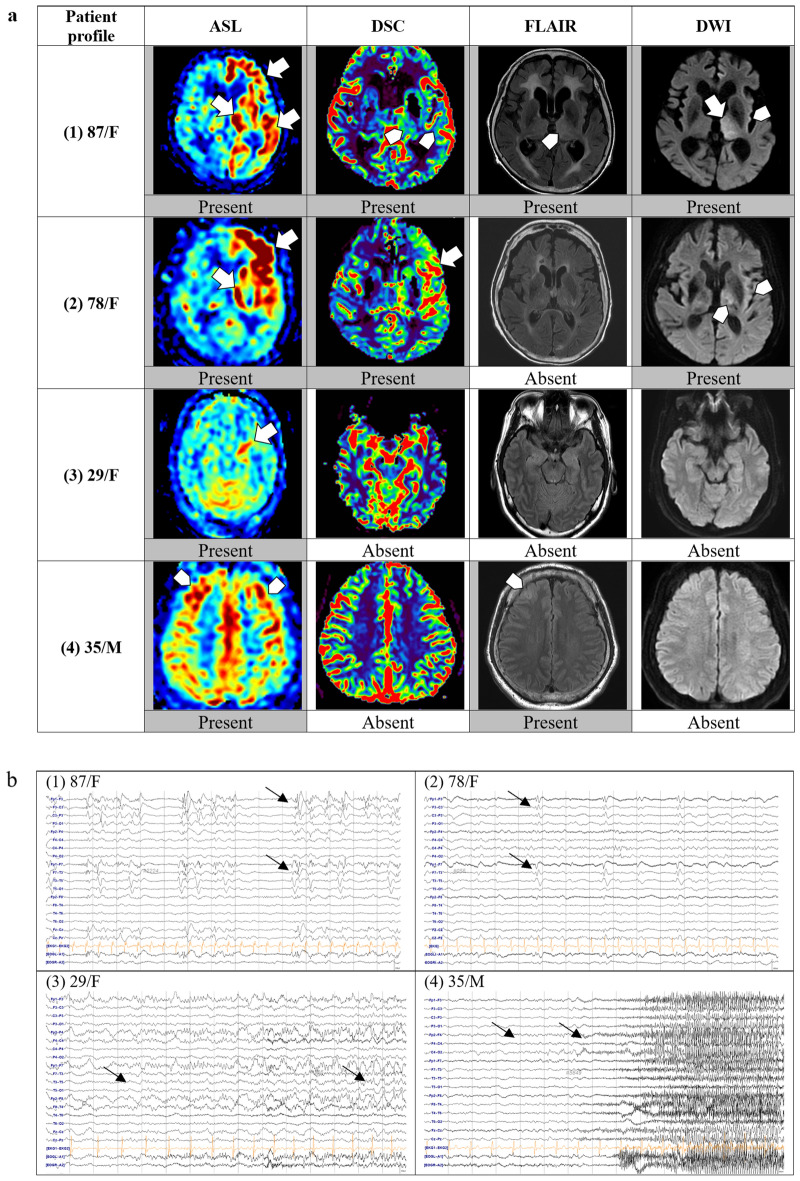
Examples of brain MRI sequences and EEGs of four representative patients with status epilepticus. Brain MRI and EEG of each patient were listed according to the same number from (1) to (4). (**a**) Brain MRI: (1) ASL showing hyperperfusion in the left frontal and temporal cortices and the left thalamus. DSC reveals subtle increases in perfusion in the left thalamus and lateral temporal lobe. FLAIR and DWI show changes in the left thalamus and insula. (2) ASL and DSC show definite hyperperfusion in the left frontal and temporal lobes, FLAIR shows no changes, and DWI shows subtle restrictions in the left thalamus and insula. (3) ASL shows definite hyperperfusion in the left hippocampus, but no signal changes are noted on DSC, FLAIR, or DWI. (4) ASL showing subtle hyperperfusion in the bilateral frontal cortices, and subtle T2 hyperintensity is noted in the left frontal cortex. (**b**) EEG: (1) Left frontotemporal periodic discharges. (2) Left frontotemporal periodic discharges. (3) Left temporal rhythmic delta activity with evolution indicating an electrographic seizure. (4) Right frontal theta rhythm evolving to beta activity, indicating an electrographic seizure. *ASL* arterial spin labeling; DSC, dynamic susceptibility contrast, *DWI* diffusion-weighted imaging, *EEG* electroencephalogram, *FLAIR* fluid-attenuated inversion recovery, *MRI* magnetic resonance imaging.

SE-related changes in signal intensity of each MRI sequence were scored as present or absent. Example cases representing each grade are illustrated in Fig. 2. During MRI review, the focus was mainly placed on brain structures where SE-related changes are predominant, including the mesial temporal lobe (hippocampus, amygdala), thalamus, insula, and lateral temporal lobe. We first identified the presence of restricted diffusion seen as hyperintensity on DWI images and hyperintense cortical edema on FLAIR. Then, perfusion images, including ASL images and relative CBF on DSC images, were visually assessed. Both hyperperfusion and hypoperfusion in a focal region were considered relevant SE-associated changes considering the underlying cause or damage found on MR images. In particular, the thalamic perfusion change was separately indicated including lateralization.

One neurologist (T.-J. Kim) and one neuroradiologist (J.W. Choi) who were initially blinded to the clinical information visually evaluated and scored the MR images. The raters scored each randomly assigned MR sequence without the patient’s clinical information, and each sequence was rated at intervals of one-week or more to avoid overestimation from the previous rating. The raters were considered to have agreed on the image evaluation if their scores were consistent on the direction of perfusion change for at least one anatomic location (frontal, temporal, mesial temporal, parietal, occipital, or thalamus). The scores were compared, and if the results differed, another neurologist (J.Y. Choi) determined the final score after a thorough discussion.

### Quantitative analysis of ASL

We measured values of regional cerebral blood flow from ASL according to the Ohtomo S et al^12^. Briefly, regions of interest (ROIs) were drawn on equivalent 10 axial planes of the ASL images. ROIs were drawn as polygons along the shape of the cortex and were drawn with the same size and consistent location in all patients. The total number of ROIs was 52 including two in the thalamus, nine in the frontal lobe, five in the temporal lobe, one in the hippocampus and four in each of the parietal and occipital lobes, in the unilateral hemisphere. Cerebral blood flow values in each lobe were averaged for further analysis. Asymmetry index was defined according to the Giovannini G et al. as below formula^38^:

$${\text{Asymmetry}}\,\, {\text{index}}\, = \,[\left| {{\rm{ROI}}right{-}{\rm{ROI}}{left}} \right|{\rm{ }}/{\rm{ }}\left( {{\rm{ROI}}right\, + \,{\rm{ROI}}left)} \right]\, \times \, 100$$

### Statistical analysis

Clinical data are presented as the mean ± standard deviation or the number with percentage. Sensitivity, specificity, positive predictive value, negative predictive value, and accuracy of each methodology and prognostic score were calculated. McNemar’s test was used to assess the statistical significance of the observed differences in method sensitivities. For group comparisons, we used the Mann–Whitney U test and Fisher’s exact test for continuous and categorical variables, respectively. Inter-rater reliability was evaluated using Cohen’s kappa coefficient. All statistical analyses were performed using IBM SPSS Statistics version 25.0 (IBM Co., Armonk, NY, USA). Differences were considered statistically significant at a two-tailed P-value of ≤ 0.05.

## Supplementary Information

Supplementary Information.

## Data Availability

All data that supports the findings of this study are included in the supplementary material of this article. Individual participant data that underlie the results reported in this article, after de-identification are available from the corresponding author upon reasonable request.
