# Supplementary material for: Usefulness of arterial spin labeling perfusion as an initial evaluation of status epilepticus
Source: Sci Rep. 2021 Dec 20;11:24218. doi: 10.1038/s41598-021-03698-7 (PMC8688435; doi:10.1038/s41598-021-03698-7)
Supplement: Supplementary file 1 — Supplementary Information. [file 41598_2021_3698_MOESM1_ESM.pdf]

Usefulness of arterial spin labeling perfusion as an initial evaluation of status epilepticus

Tae-Joon Kim[1,*], Jin Wook Choi[2,*], Miran Han[2], Byung Gon Kim[1,3], Sun Ah Park[1,4], Kyoon Huh[1,5], Jun Young Choi[1,3,¶]

[1]Department of Neurology, Ajou University School of Medicine, Suwon, Republic of Korea

[2]Department of Radiology, Ajou University School of Medicine, Suwon, Republic of Korea

[3]Department of Brain Science, Ajou University School of Medicine, Suwon, Republic of Korea

[4]Department of Anatomy, Ajou University School of Medicine, Suwon, Republic of Korea

[5]Department of Medical Humanities & Social Medicine, Ajou University School of Medicine, Suwon, Republic of Korea

[*]Co-first authors; these two authors contributed equally to the manuscript.

[¶]Corresponding author.

Jun Young Choi, MD, PhD.

Departments of Brain Science and Neurology, Ajou University School of Medicine, 164, World cup-ro, Yeongtong-gu, Suwon, Gyeonggi-do, 16499, Republic of Korea.

Tel: +82-31-219-4544, Fax: +82-31-219-4530

E-mail: jychoi@aumc.ac.kr, taz312@gmail.com

Supplementary Table S1. Detailed description of clinical information of 51 patients with SE

Pt. no.	Age	Sex	SE etiology	Seizure pattern	Time progress (BZD, AED, MRI, EEG / anesthesia)* in hour	Change of each MRI sequence (ASL, DSC, FLAIR, DWI)	Angiography	ASL	EEG	Match between EEG and ASL	Refractoriness	Discharge mRS
**1**	59	M	Alcohol withdrawal provoked combined with traumatic SAH, SDH	GCSE only	0.6, 2.3, 9.9, 11.8	↑↑↑ -	no steno-occlusion	Rt hemisphere with thalamus	Rt F-T ED	Lobar		2
**2**	53	M	Unknown, possible autoimmune	SPSE evolving into GCSE	1.3, 43.4, 6.6, 17.2 / 64.2	↑↑↑↑	no steno-occlusion	Bilateral cingulate	Bi LPD, Lt T ictal	Lobar	Super-RSE	6
**3**	20	M	Metabolic, hypoglycemic	GCSE only	0.8, 7.1, 9.8, 23.3	- - - -	n/a	no change	nonspecific	nonspecific, both		1
**4**	82	M	Posterior reversible encephalopathy syndrome	SPSE	3.5, 5.6, 7.8, 4.5 / 26.5	↑↑↑↑	no steno-occlusion	Rt T and thalamus	Rt T ictal & LPD	Lobar	Super-RSE	5
**5**	53	M	Metabolic, recurrent hypoglycemic, fever	NCSE	0.6, 17.3, 17.7, 14.7	↑↑ - -	Mild basilar artery stenosis	Rt hemisphere	Rt hemisphere ictal & LPD	Lobar		2
**6**	69	M	Acute Rt basal ganglia infarction with previous stroke	GCSE evolving into NCSE	10.3, 13.5, 17.6, 21.1	↑ - - -	Lt ICA occlusion, Rt dICA & MCA severe stenosis	Rt cingulate and thalamus	nonspecific	nonspecific EEG		6
**7**	77	M	Old cerebral infarction	GCSE evolving into NCSE	0.7, 1.2, 5.1, 7.6	↑↑↑ -	Rt pICA 80% stenosis, Lt pICA 50% stenosis, bi MCA moderate stenosis, Rt VA severe stenosis	Rt F-T	Rt F-T ictal & LPD	Lobar	RSE	4
**8**	60	F	Old cerebral infarction	GCSE only	1.8, 2.6, 11.1, 4.7	↑↑↑↑	Rt ACA & MCA severe stenoses, Lt pICA mild stenosis, Lt PCA narrowing	Rt T-O	Rt T-O ictal	Lobar		5
**9**	51	F	Old traumatic SAH	SPSE evolving into GCSE	1.3, 21.0, 14.6, 18.5	↑↑ - ↑	Bi carotid bulb mild stenosis	Lt hemisphere with Lt thalamus	Lt F-T ictal, Lt LPD	Lobar	RSE	2
**10**	30	M	AED dose decreased and skipped once, remote methotrexate induced encephalopathy	GCSE only	0.7, 4.4, 4.0, 44.0	↓↓↑↑	no steno-occlusion	Rt F-T-P hypoperfusion	Rt T ED	Lobar, ASL hypoperfusion		1
**11**	83	M	Dementia	GCSE only	0.7, 2.0, 3.7, 10.9	↓ - - -	Rt dICA severe stenosis, multifocal stenoses at intracranial arteries	Lt hemispheric and thalamic hypoperfusion	Lt F-T ED	Lobar, ASL hypoperfusion		2
**12**	79	M	Old Rt T ICH, traumatic	GCSE evolving into NCSE	0.7, 3.8, 6.6, 14.5	↑ - ↑ -	n/a	Rt insula and medial T and both thalamus	Rt T ictal & LPD	Lobar	RSE	2
**13**	63	M	AED skipped for one month, old Rt MCA territory infarction	SPSE	37.3, 38.4, 46.3, 50.9	- - ↑ -	n/a	no change	Rt F-T ictal	nonspecific ASL	RSE	3
**14**	21	M	Unknown, possible autoimmune	GCSE only	0.5, 4.5, 14.6, 18.7 / 7.0	↑ - ↑ -	no steno-occlusion	Rt hemisphere	Rt T-O > Lt T ictal	Lobar	Super-RSE	6
**15**	61	M	Old Lt MCA territory infarction	GCSE only	0.4, 4.8, 6.5, 18.9	↓↓↑ -	no steno-occlusion	Lt hemispheric and thalamic hypoperfusion	Lt hemisphere, postictal	Lobar, ASL hypoperfusion		2
**16**	45	F	AED change in known epilepsy	GCSE only	1.1, 2.4, 5.6, 13.4	- - ↑ -	n/a	no change	Rt T ED	nonspecific ASL		1

**17**	74	M	Known epilepsy, old Rt contusion hemorrhage	GCSE only	1.8, 4.4, 22.4, 4.7	↑ - ↑ -	Lt carotid bulb mild stenosis	Rt hemisphere	nonspecific	nonspecific EEG		2
**18**	87	F	Known epilepsy, old Rt posterior cerebromalacia	GCSE only	0.3, 3.1, 5.3, 9.9	↓ - ↑ -	multifocal stenoses including bi MCA and ACA	Rt hemispheric and thalamic hypoperfusion	Rt hemisphere, postictal	Lobar, ASL hypoperfusion		4
**19**	60	M	Old Lt T neurocysticercosis	GCSE only	0.7, 42.0, 9.1, 15.6	↑ - ↑ -	n/a	Lt T and thalamus	nonspecific	nonspecific EEG		0
**20**	62	F	Old Lt traumatic SDH	NCSE	3.4, 7.8, 11.0, 45.2	↑ - - -	no steno-occlusion	Rt hemisphere	Lt F-T ictal, bi hemispheric ED	Discordant		3
**21**	76	F	Old cerebral infarction	GCSE only	0.2, 9.2, 8.7, 12.8	↑ ↑ - -	Lt MCA and Lt VA stenosis	Rt T-O and thalamus	Bi hemisphere LPD	Discordant		4
**22**	78	M	Old ICH	GCSE only	1.3, 6.5, 17.4, 19.7	↑ ↑ ↑ -	Lt MCA, Lt VA, Rt pICA moderate stenosis	Rt T-P-O	Rt hemisphere, postictal	Lobar		4
**23**	82	M	Paraneoplastic syndrome (anti-hu)	GCSE only	0.3, 8.2, 6.6, 14.0	↑ ↑ - -	no steno-occlusion	Rt T-O-F and thalamus	Rt hemispheric RD, Rt P ED	Lobar		1
**24**	36	M	Old traumatic ICH	GCSE only	1.3, 5.3, 6.2, 10.9	↑ - ↑ -	n/a	Rt T-O	Rt F-T ED	Lobar		0
**25**	78	M	Old Lt F ICH	GCSE evolving into NCSE	4.0, 11.6, 14.3, 29.7	↑ ↑ ↑ ↑	n/a	Lt F and both thalamus	Lt F-T ictal	Lobar	RSE	2
**26**	84	F	Old Rt MCA territory infarction	GCSE only	0.6, 5.0, 6.0, 12.1	↑ - ↑ ↑	Bi MCA focal stenoses	Rt T	Rt F postictal	Laterality		2
**27**	54	M	Old traumatic bi F SDH	GCSE only	0.8, 6.1, 6.7, 13.5	↑ - ↑ -	Lt carotid bulb mild stenosis	Lt medial T and both thalamus	nonspecific	nonspecific EEG		0
**28**	68	M	Cancer brain metastasis	GCSE only	1.3, 2.3, 2.9, 6.0	↑ ↑ ↑ ↑	no steno-occlusion	Bilateral F and Lt thalamus	nonspecific	nonspecific EEG		6
**29**	85	M	Vascular dementia with cerebral amyloid angiopathy	GCSE only	1.0, 5.9, 8.9, 12.4	↑ - ↑ ↑	n/a	Lt medial T	nonspecific	nonspecific EEG	RSE	2
**30**	29	F	Unknown, possible autoimmune	GCSE evolving into NCSE	2.5, 27.2, 15.0, 19.9	↑ - ↑ -	n/a	Lt medial T	Bi (Lt>Rt) T ictal	Lobar		1
**31**	78	F	Recurrent fever of unknown origin	SPSE	2.8, 6.7, 6.2, 10.3	↑ ↑ - ↑	no steno-occlusion	Rt F-T and Lt thalamus	Lt F-T LPD	Discordant		5
**32**	84	F	Hyponatremia with old cerebral infarction	GCSE evolving into NCSE	1.0, 23.8, 18.9, 28.9 / 32.4	↑ ↑ - ↑	no steno-occlusion	Lt P	Lt F-P ictal	Lobar	RSE	5
**33**	77	M	Acute hypoxia	GCSE only	0.9, 1.2, 4.3, 10.1	↑ ↑ - -	n/a	Rt hemisphere with thalamus	Lt F-T LPD	Discordant		2
**34**	75	M	Alzheimer’s dementia	GCSE only	1.2, 1.7, 5.5, 9.3	↑ - - -	Lt pICA 50% stenosis, Rt pICA 30% stenosis, BA stenosis, Rt VA occlusion	Lt lateral T	nonspecific	nonspecific EEG		4
**35**	61	F	Unknown, possible autoimmune	GCSE evolving into NCSE	1.5, 13.8, 16.3, 8.1 / 79.7	- - - -	n/a	no change	Bi T ictal	nonspecific ASL	Super-RSE	2
**36**	84	M	Old Rt MCA territory infarction	GCSE only	0.8, 1.4, 2.7, 6.3	↑ - - -	Bi MCA diffuse narrowing, bi PCA mild stenosis	Lt medial F and thalamus	Lt F-T ictal	Lobar		5
**37**	47	M	Propofol induced	NCSE	29.8, 40.0, 25.9, 29.5	↑ ↑ - -	no steno-occlusion	Lt F-P	Lt F postictal	Lobar		3
**38**	81	M	Vascular dementia	GCSE evolving into NCSE	0.5, 4.8, 8.1, 20.9 / 39.0	↑ - ↑ ↑	no steno-occlusion	Rt medial T and thalamus	Rt F ictal	Laterality	RSE	6
**39**	56	M	AED skipped, chronic alcoholic	GCSE evolving into NCSE	0.9, 4.8, 12.8, 15.9 / 81.6	↑ - ↑ ↑	no steno-occlusion	Lt T	Lt T-P LPD	Lobar	Super-RSE	4
**40**	87	F	Vascular dementia	GCSE evolving into NCSE	0.6, 1.5, 2.9, 9.0	↑ ↑ ↑ ↑	no steno-occlusion	Lt hemisphere with thalamus	Lt hemispheric LPD	Lobar	RSE	6
**41**	35	M	Tuberous sclerosis	GCSE only	3.7, 4.3, 5.7, 12.3	↑ - ↑ -	n/a	Bi F	Bi F ictal & ED	Lobar	RSE	1

**42**	68	M	Bain tumor	GCSE evolving into NCSE	3.3, 4.9, 6.6, 11.0	↑ - - ↑	n/a	Rt medial T and thalamus	Rt F LPD	Laterality	RSE	4
**43**	55	M	Known epilepsy	NCSE	7.5, 7.5, 23.4, 18.0	↑ - - -	n/a	Rt F-T	Rt F ictal	Lobar	RSE	2
**44**	62	M	Alzheimer's dementia	GCSE only	3.2, 3.1, 20.4, 36.3	↑ - - -	Bi pICA severe stenoses (Rt 90%, Lt 70%)	Rt P	Rt T ictal	Laterality	RSE	3
**45**	79	M	Alzheimer's dementia	GCSE evolving into NCSE	22.5, 22.5, 11.7, 8.0	↑ - - -	n/a	Rt medial T	Rt T ED	Lobar	RSE	5
**46**	34	F	MELAS syndrome	SPSE	3.9, 3.0, 9.4, 13.8	↑ ↑ ↑ ↑	no steno-occlusion	Rt P-T-O and thalamus	Rt T ictal	Lobar		2
**47**	19	F	Autoimmune encephalitis (anti-NMDAR)	GCSE only	2.1, 3.3, 13.7, 112.2	↑ - - -	n/a	Rt hemisphere	Bi T (Rt>Lt) postictal	Lobar		2
**48**	78	F	Old cerebral infarction	SPSE evolving into GCSE	2.8, 48.8, 2.4, 26.6	↑ - - ↑	no steno-occlusion	Lt hemisphere with thalamus	Lt T postictal	Lobar		1
**49**	57	M	Old brain hemorrhage	GCSE evolving into NCSE	0.6, 2.3, 5.9, 10.0	↑ - - ↑	no steno-occlusion	Lt lateral T and thalamus	Lt T RD	Lobar		1
**50**	50	M	Old cerebromalacia	GCSE only	1.6, 1.8, 13.3, 3.0	- - - -	no steno-occlusion	no change	Lt F-T postictal	nonspecific ASL		1
**51**	59	F	Unknown	GCSE only	1.4, 3.9, 6.8, 21.7	↑ - - -	no steno-occlusion	Lt T	Lt T postictal	Lobar		3

*Time progress of FAT to BZD, AED, MRI, EEG / anesthesia. Only in the case of patients who were treated with continuous infusion of anesthetic agents for refractory status epilepticus, the timepoint of anesthesia is indicated.

Abbreviations: SE, status epilepticus; BZD, benzodiazepine; AED, antiepileptic drug; MRI, magnetic resonance imaging; EEG, electroencephalography; ASL, arterial spin labeling; DSC, dynamic susceptibility contrast; FLAIR, fluid attenuated inversion recovery; DWI, diffusion weighted imaging; mRS, modified Rankin Scale; SAH, subarachnoid hemorrhage; SDH, subdural hematoma; ICH, intracerebral hemorrhage; MCA, middle cerebral artery; NMDAR, N-methyl-D-aspartate receptor; GCSE, generalized convulsive SE; SPS, simple partial SE; NCSE, nonconvulsive SE; n/a, not available; ICA, internal carotid artery; dICA, distal internal carotid artery; pICA, proximal internal carotid artery; ACA, anterior cerebral artery; PCA, posterior cerebral artery; VA, vertebral artery; F, frontal lobe; T, temporal lobe; O, occipital lobe; Lt, left; Rt, right; Bi, bilateral; LPD, lateralized periodic discharge; ED, epileptiform discharge; RSE, refractory SE.

SUPPLEMENTARY FIGURE

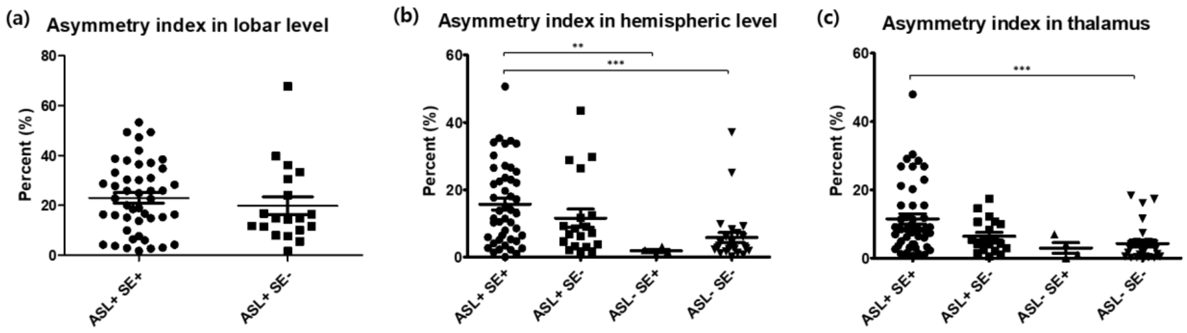

Supplementary Figure 1. Quantitative analysis of ASL perfusion image. Asymmetric indices (a) between lesion (hyper- or hypoperfusion) and non-lesion sides lobar level, (b) between both hemispheres, and (c) between both thalami. Abbreviations: ASL, arterial spin labeling; ASL+, visual change present in ASL; ASL-, visual change absent in ASL; SE+, group with status epilepticus; SE-, group with self-limited seizures
